# Supplementary material for: Disability and the achievement of Universal Health Coverage in the Maldives
Source: PLoS One. 2022 Dec 21;17(12):e0278292. doi: 10.1371/journal.pone.0278292 (PMC9770361; doi:10.1371/journal.pone.0278292)
Supplement: S1 Table — (DOCX) [file pone.0278292.s001.docx]

**S1 Table. Predictors of reporting a need for an assistive device or specialist service among people with disabilities**
